# Plant Growth-Promoting and Herbicidal Bacteria as Potential Bio-Based Solutions for Agriculture in Desertic Regions

**DOI:** 10.3390/plants14010009

**Published:** 2024-12-24

**Authors:** Patricio Muñoz-Torres, Wilson Huanca-Mamani, Steffany Cárdenas-Ninasivincha, Yola Aguilar, Antonio Quezada, Franco Bugueño

**Affiliations:** Laboratory of Plant Pathology and Bioproducts, Faculty of Agronomic Sciences, University of Tarapacá, Av. General Velásquez 1775, Arica 1000000, Chile; whuanca@uta.cl (W.H.-M.); sfcninasivincha@gmail.com (S.C.-N.); catacorayol35@gmail.com (Y.A.); quezadaortega@gmail.com (A.Q.); franco.bugueno.guerrero@gmail.com (F.B.)

**Keywords:** plant-growth promoting bacteria, biocontrol, Atacama Desert, desert agriculture, salinity, hot springs

## Abstract

The region of Arica and Parinacota hosts unexplored remote sites with unique characteristics suitable for developing novel agricultural bioproducts. Notable locations include Jurasi Hot Springs, Polloquere Hot Springs, and Amuyo Lagoons, featuring open pools fed by thermal mountain springs. These geothermal sites harbor bacteria with plant growth-promoting activities, particularly interesting to the strains J19, TP22, A20, and A3. These bacteria possess in vitro plant growth-promoting traits, the ability to produce hydrolytic enzymes, and the capacity to inhibit phytopathogenic fungi. Moreover, they can tolerate different concentrations of NaCl and boron, making them suitable for developing new agricultural bioproducts for arid environments. The bacterial strains A3 and A20 have a positive effect on the growth of the aerial part of tomato plants (increased stem length, fresh and dry weight), with a significant increment in proline concentration and chlorophyll A and B content under saline conditions. Meanwhile, the strains J19 and TP22 exhibit herbicidal activity against *Cenchrus echinatus* by reducing root elongation and germination of the weed. These strains possess plant growth-promoting traits and improve plant resistance to salinity stress. They are promising candidates for developing innovative bio-based agricultural products suited to arid and semi-arid regions.

## 1. Introduction

Global food demand is a complex and growing challenge driven by various factors, including population growth, climate change, changing consumption patterns, and urbanization. As the world population approaches the projected 10 billion people by 2050, food demand is expected to increase by 50% compared to current levels; this increase generates significant challenges for agronomic production systems since they will have to produce more food in the context of limited natural resources and under increasing environmental pressure [1]. Additionally, arid and semi-arid regions cover approximately 66.7 million km² of the Earth’s surface, hosting around 2 billion people. These regions are expanding due to climate change, posing significant threats to ecosystems and livelihoods. Key challenges include desertification caused by severe soil degradation from climatic changes and human activities, resulting in soil erosion, low nutrient levels, and reduced vegetation cover. These challenges endanger food security, force migration, and reduce biodiversity. The harsh environmental conditions in these regions are characterized by poor soil structure, low organic matter, high salinity, water scarcity, and extreme temperatures. These factors limit soil fertility, reduce water-holding capacity, increase greenhouse gas emissions, and exacerbate soil degradation processes such as erosion and biodiversity loss [2].

Additionally, microbial activity critical for nutrient cycling is compromised, leading to ecosystems that are highly sensitive to disturbances and slow to recover. Sustainable approaches that increase crop productivity while preserving ecological balance are urgently needed to address these problems. These approaches involve developing innovative methods to rehabilitate degraded lands and mitigate abiotic stresses in crops without compromising soil health and ecosystem functions [2].

In Chile, agriculture is a paramount activity. The production of vegetables in the Arica and Parinacota Region (Atacama Desert) is vital since it supplies the country during winter [3]. Tomato (*Solanum lycopersicum* L.) is one of the most cultivated and important horticultural species in the country. Its main production destinations are fresh consumption and industrial purposes [3]. In particular, in the Arica and Parinacota Region, tomatoes are one of the most imperative horticultural species for fresh consumption. Tomato production reached a cultivated area of ~840 ha during 2019, of which 83% was an outdoor farming system and 17% was greenhouse cultivation [4]. The recent technological input and cultivation expansion in this region are worth mentioning. The tomato cultivation has incorporated new technologies, including innovations in irrigation systems, the use of anti-vector meshes, and the incorporation of grafted plants in invigorating patterns [5]. These technologies improved tomato production in the Atacama Desert.

It is essential to mention that the agriculture of the Arica and Parinacota Region is carried out under extreme conditions of cultivation, including aridity, high concentrations of salts and boron, a comprehensive thermal amplitude between the day and night, poor soil composition, and high UV radiation, among other conditions that are restrictive for agricultural activities in arid and semiarid regions [6,7]. Under this scenario, commercial agricultural bioproducts show erratic behavior because the microorganisms formulated in these types of products are naturally adapted to be active and viable in sites that possess similar conditions to those of the microorganism obtained. For example, commercial bioproducts based on the fungus *Trichoderma* spp. show inconsistent results when applied as biocontrol agents to prevent infectious diseases in northern Chile, where salinity and the presence of boron limit the growth and sporulation of the fungus, reducing its usefulness as a biocontrol agent [8]. From this perspective, there is a noticeable shortage of commercial bioproducts tailored for extreme environments. Consequently, one strategy to develop new agricultural bioproducts could involve bioprospecting in extreme habitats, where native microorganisms (naturally adapted to harsh conditions) may serve as innovative biofertilizers, biostimulants, and biocontrol agents.

Extreme environments are habitats defined by environmental conditions surpassing the optimal human development limits. These include extremes in pH (acidic or alkaline), temperature (either high or low), saturating salinity levels, intense radiation, and elevated pressures. Furthermore, these environments often exhibit seasonal fluctuations in extreme conditions, supporting the growth of both extremophilic and extremotolerant microorganisms. Microorganisms inhabiting these environments are attracting increased interest due to their unique biodiversity, evolutionary adaptations, and potential for biotechnological applications [9].

Among the applications of extremotolerants, the use of these microorganisms as plant growth promoters has been described. For example, Kumar et al. [10] studied the plant growth promotion (PGP) activity of 53 bacteria isolated from the Jakrem hot spring (India) that belong to the *Bacillus* genus. The authors observed that 37.7% of bacterial strains exhibited PGP and hydrolytic activities, and the strains JAB1, JAB8, and JAB100 were particularly interesting. The combination of these bacterial strains significantly enhanced the growth and development of *Brassica juncea* plants, as evidenced by the increase in shoot and root length, fresh and dry weight, and higher levels of proteins, phenol, flavonoid, and chlorophyll contents compared to the control; this highlights the potential of extremotolerant bacteria to serve as bioinoculants, promoting crop productivity within sustainable agricultural practices. Additionally, Tuesta-Popolizio et al. [11] successfully isolated, identified, and characterized 15 novel microbial strains from the Los Negritos geothermal site in Mexico. These strains demonstrated plant growth-promoting activities, including auxin production, inorganic phosphate solubilization, and siderophore production. This information indicates that geothermal sites could be valuable sources for discovering bacteria that enhance plant growth.

There are several geothermal sites located in the Arica and Parinacota Region. Jurasi Hot Springs, Polloquere Hot Springs, and Amuyo Lagoons are among them. They constitute a series of open pools supplied by thermal waters emerging from the surrounding mountains. Analysis of water and sediments reveals that these three sites are characterized by high contents of salts (particularly Na^+^, Ca^2+^, Cl^−^, and SO_4_^2−^), boron, and arsenic [12], similar to the cultivation conditions of the Arica and Parinacota Region. In this context, it can be assumed that bacteria isolated from Jurasi Hot Springs, Amuyo Lagoons, and Polloquere Hot Springs are naturally adapted to the saline-boric conditions characteristic of these environments. This adaptation may support the development of new bio-based products for agriculture conducted in arid conditions.

Previously, we have isolated 57 bacteria from these three geothermal sites. Sequence analysis indicated that these bacteria belong to several genera, including *Bacillus* and *Pseudomonas*. The functional characterization indicated the presence of PGP traits, hydrolytic enzymes, and biocontrol activity against phytopathogenic fungi, revealing the potential to develop new bio-based products for agriculture in desertic conditions [12].

This study aimed to identify and perform functional characterization in vitro and in planta of four bacterial strains (J19, TP22, A20, and A3) sourced from Jurasi Hot Springs, Polloquere Hot Springs, and Amuyo Lagoons. These strains were selected from the bacterial culture collection of the Laboratory of Plant Pathology and Bioproducts at Universidad de Tarapacá (Chile) due to their promising plant growth-promoting (PGP) activities, positioning them as excellent candidates for developing novel bioproducts. The need to develop sustainable agriculture with a reduced load of agrochemicals, which could be replaced by biological alternatives, being of particular interest bioproducts adapted to specific environments, is of utmost importance. Moreover, it is of particular interest to promote the innocuous production of fruits and vegetables in an accelerated climate change, where arid and semi-arid sites are gaining more importance, being necessary to develop new active and viable bioproducts for the cultivation carried out in desertic conditions. In this sense, using the strains J19, TP22, A20, and A3 could represent an alternative for developing new bio-based agricultural products.

## 2. Results

### 2.1. Strains Characterization and Identification

Four strains were selected from the bacterial culture collection of the Laboratory of Plant Pathology and Bioproducts belonging to the Universidad de Tarapacá due to their promising in vitro PGP activities [12]. The strains corresponded to isolates J19, TP22, A20, and A3 (Table 1).

The J19 strain was isolated from the Jurasi Hot Springs, and it is a member of the *Pseudomonas* genus, showing 99.93% similarity to *Pseudomonas aeruginosa* (NR_117678.1). Microbiological characterization (Table 1) showed Gram-negative straight rods with motility in a semi-solid medium, producing acids from D-glucose and D-galactose. However, no acid production was detected when L-asparagine, D-cellobiose, D-melibiose, D-fructose, D-rhamnose, D-raffinose, D-mannitol, D-sorbitol, D-inositol, sucrose, and lactose were used as carbon sources.

The TP22 strain was obtained from the Polloquere Hot Springs and belongs to the *Bacillus* genus, having 99.92% similarity to *Bacillus velezensis* (NR_075005.2). The microbiological characterization revealed the presence of Gram-positive and motile straight rods, which produced acids from D-glucose, D-cellobiose, D-raffinose, D-mannitol, D-sorbitol, sucrose, and lactose. No acid production was detected when L-asparagine, D-galactose, D-melibiose, D-fructose, D-rhamnose, and D-inositol were used as carbon sources.

The strain A20 was isolated from Amuyo Lagoons and is a member of the *Pseudomonas* genus. It shows 99.80% similarity to the *Pseudomonas rhodesiae* (NR_024911.1). Its microbiological characterization showed the presence of Gram-negative and non-motile straight rods. This strain produced acids from D-glucose, D-galactose, D-cellobiose, D-melibiose, D-fructose, D-rhamnose, and D-mannitol; however, no acids were produced when L-asparagine, D-raffinose, D-sorbitol, D-inositol, sucrose, and lactose were used as carbon sources.

The strain A3 was isolated from Amuyo Lagoons and is a member of the *Bacillus* genus, 99.81% similar to *Bacillus sanguinis* (NR_175555.1). Microbiological characterization showed Gram-positive and motile straight rods, which produce acids from D-cellobiose and D-fructose. However, no acid production was detected when L-asparagine, D-glucose, D-galactose, D-melibiose, D-rhamnose, D-raffinose, D-mannitol, D-sorbitol, D-inositol, sucrose, and lactose were used as carbon sources.

Similarity results indicate that the strains J19 and A20 belong to the genus *Pseudomonas*, showing close relationships with *P. aeruginosa* and *P. rhodesiae*, respectively. In contrast, strains TP22 and A3 are members of the genus *Bacillus*, closely related to *B. velezensis* and *B. sanguinis*, respectively.

The diffusion method performed antibiotic susceptibility of the four strains (Table 2). The strain J19 was sensitive to ciprofloxacin, having intermediate susceptibility to chloramphenicol. The strains TP22 and A20 were sensitive to chloramphenicol, ciprofloxacin, kanamycin, and neomycin. Meanwhile, strain A3 was sensitive to chloramphenicol and kanamycin, having intermediate susceptibility to ciprofloxacin and neomycin.

### 2.2. Tolerance of Bacterial Strains to NaCl and H_3_BO_3_

A quantitative methodology based on measuring optical density to 600 nm (OD_600_) was performed to determine the tolerance of the four strains to saline-boric conditions (Table 3); this established that the control condition corresponded to the liquid medium (King’s medium B) without the presence of NaCl and H_3_BO_3_ (abundant growth).

For the strain J19, abundant growth was observed in a liquid medium supplemented with 8 g/L and 15 g/L of NaCl or 10 ppm of H_3_BO_3_. However, a slight decrease in the OD_600_ was observed when 30 g/L of NaCl or 100–300 ppm of H_3_BO_3_ were used to supplement the King’s medium B. When the strain was grown in a liquid medium amended with 0.86 g/L NaCl and 114 ppm H_3_BO_3_ (1× concentration of NaCl and H_3_BO_3_ from Lluta irrigation water), no significant differences were observed in comparison to the control conditions, even when the concentration of both compounds was raised up 8×.

When the strain TP22 was grown in the liquid medium supplemented with 8 g/L and 15 g/L of NaCl or 10–300 ppm of H_3_BO_3_, abundant growth was measured. A similar effect was observed when the medium was amended with 1×–8× Lluta irrigation water. A decrement in the OD_600_ was observed only at 30 g/L of NaCl.

No significant differences were measured when strain A20 was grown in King’s medium B supplemented with 8–30 g/L of NaCl or 10 ppm of H_3_BO_3_. An inhibitory effect on the growth of strain A20 was observed at higher H_3_BO_3_ concentrations. Furthermore, a slight increment in the growth of the bacterium A20 was measured at 1×–4× Lluta irrigation water, with an inhibitory effect at higher concentrations.

For strain A3, no significant differences were observed in the bacterial growth at the different concentrations of NaCl and H_3_BO_3_ employed, even in the presence of combinations of both compounds.

### 2.3. In Vitro PGP Activities

In vitro PGP activities of the strains J19, TP22, A20, and A3 are described in Table 4. The strain J19 exhibited significant plant growth-promoting activities, including nitrogen fixation, phosphate solubilization, and production of siderophores, auxins, ACC deaminase, and HCN. Additionally, it produced enzymes, including protease, cellulase, and lipase, enabling the degradation of proteins, cellulose, and lipids, respectively. Moreover, this strain was able to inhibit the growth of all tested phytopathogenic fungi (*Botrytis cinerea*, *Fusarium oxysporum*, *Geotrichum candidum*, *Macrophomina phaseolina*, and *Monilinia fructicola*).

The strain TP22 demonstrated the ability to fix elemental nitrogen and produced protease and chitinase, facilitating protein and chitin degradation, respectively. The biocontrol assay against phytopathogenic fungi revealed that this bacterium can inhibit the growth of *B. cinerea*, *F. oxysporum*, *G. candidum*, *M. phaseolina*, and *M. fructicola*.

Meanwhile, the strains A20 and A3 showed positive results for auxin production and phosphate solubilization on PVK solid medium, as indicated by clear halos surrounding their colonies. The strain A20 demonstrated nitrogen-fixing ability in a semisolid NFb medium, forming a subsurface film and producing ACC deaminase. While both strains did not produce siderophores, strain A3 lacked nitrogen-fixing and ACC deaminase activities. Enzymatic activity analysis revealed that the A3 strain produced protease, lipase, and cellulase but was negative for chitinase production. In contrast, the A20 strain produced protease, lipase, and cellulase but did not produce chitinase. A3 strain showed biocontrol activity against *B. cinerea*, *G. candidum*, *M. phaseolina*, and *M. fructicola*. No biocontrol activities were determined for strain A20.

### 2.4. Optimization of Bacterial Growth

**Figure 1:**
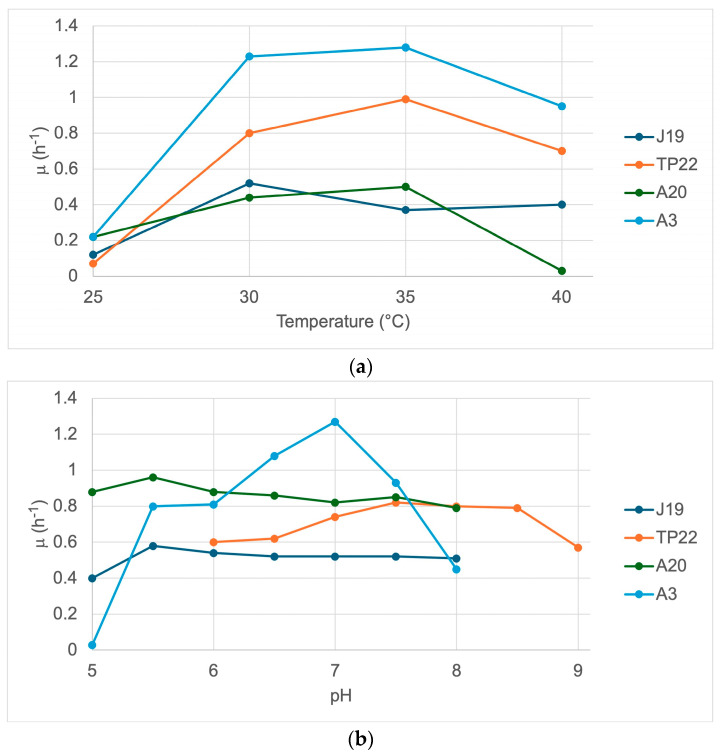

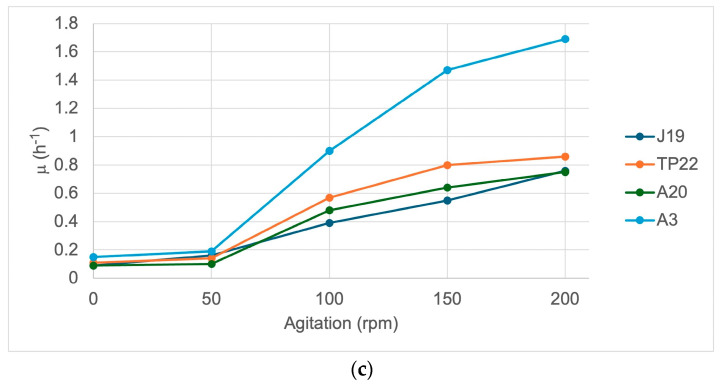
Determination of the optimal conditions for the growth of the strains J19, TP22, A20, and A3. The microbial growth rates were determined as µ (h^−1^): (**a**) temperature optimization; (**b**) pH optimization; and (**c**) agitation optimization.

Three parameters (temperature, pH, and agitation) were independently modified to optimize the bacterial growth of strains J19, TP22, A20, and A3 (Figure 1). For this purpose, the microbial growth rate (μ) was determined from each bacterium’s exponential growth phase under each condition. The highest μ value is indicative of the optimum condition.

The optimum temperature for the strain J19 was 30 °C (μ = 0.52 h^−1^); meanwhile, for the strains TP22 (μ = 0.99 h^−1^), A20 (μ = 0.50 h^−1^), and A3 (μ = 1.28 h^−1^), it was 35 °C. The optimum pH for the strain J19 was 5.5 (μ = 0.58 h^−1^), for TP22 it was 7.5 (μ = 0.82 h^−1^), for A20 it was 5.5 (μ = 0.96 h^−1^), and for A3 it was 7.0 (μ = 1.27 h^−1^). The optimum agitation for the four bacteria was 200 rpm (μ J19 = 0.76 h^−1^; μ TP22 = 0.86 h^−1^; μ A20 = 0.75 h^−1^; and μ A3 = 1.69 h^−1^).

**Figure 2:**
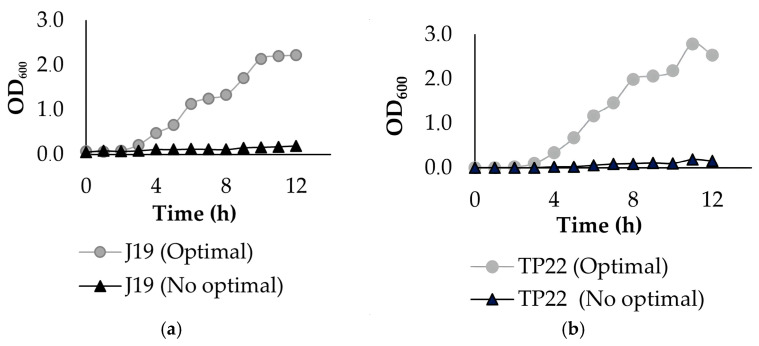

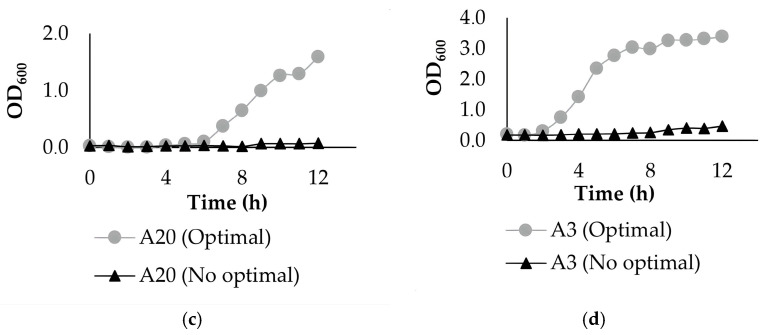
Bacterial growth curves under optimal (circle) and non-optimal (triangle) conditions: (**a**) strain J19; (**b**) strain TP22; (**c**) strain A20; (**d**) strain A3.

Bacterial growth curves were determined using the OD_600_ under optimal and non-optimal conditions (Figure 2). Under optimal conditions, the strain J19 exhibited an increase in growth (exponential phase) after 4 h of incubation, reaching its stationary phase after 10 h of post-inoculation (30 °C, 200 rpm, and pH 5.5). For strain TP22, the exponential phase began after 4 h of incubation, with the stationary phase reaching 11 h under optimal conditions (35 °C, 200 rpm, and pH 7.5). The strain A20, under optimal conditions (35 °C, 200 rpm, and pH 5.5), entered the exponential phase after 7 h of incubation, reaching the stationary phase after 12 h. The strain A3 reached the exponential phase at 3 h and the stationary phase at 9 h under optimal conditions of 35 °C, 200 rpm, and pH 7.0. However, in non-optimized conditions, the growth curves of the strains showed a lag phase between 0 and 12 h.

### 2.5. PGP Activity of the Strains J19, TP22, A20 and A3 on Tomato Plants cv. Poncho Negro

The ability of strains J19, TP22, A2,0 and A3 to promote plant growth was tested using tomato plants cv. Poncho Negro, which were inoculated with fresh 1 × 10^8^ CFU and compared with uninoculated plants (T0) and the commercial product BacNorte. The assays were conducted under standard (absent of NaCl) and saline (0.1 M NaCl) conditions (Table 5, Table 6, Table 7 and Table 8).

When tomato plants cv. Poncho Negro were inoculated with the strain J19 (Table 5), only a significant increase in aerial length was observed in comparison to T0 under saline conditions. Under standard and saline conditions, no significant differences were observed in root length, root fresh weight, root dry weight, aerial fresh weight, and aerial dry weight.

A significant increase in the root fresh weight and aerial fresh/dry weight was observed when tomato plants cv. Poncho Negro were inoculated with the strain TP22 in comparison to T0 under saline conditions (Table 6).

The inoculation of the strain A20 has a positive effect on the growth of the aerial part of tomato plants cv. Poncho Negro under saline conditions (stem length, fresh/dry weight) in comparison to T0 (Table 7).

The inoculation with the A3 strain enhances the growth of the aerial parts of tomato plants cv. Poncho Negro under saline conditions, promoting increased stem length as well as fresh and dry weight (Table 8).

### 2.6. Effect of the Inoculation of Strains J19, TP22, A20 and A3 on the Proline and Chlorophyll Content of Tomato Plants cv. Poncho Negro

Additionally, proline, chlorophyll A, and chlorophyll B contents were measured to determine the effect of the bacterial inoculation in tomato plants cv. Poncho Negro. There is a significant increase in proline concentration in tomato plants cv. Poncho Negro due to the application of BacNorte, TP22, A3, or A20 strains under saline conditions (Table 9). Moreover, there is a significant increase in chlorophyll A concentration in tomato plants cv. Poncho Negro due to the application of BacNorte, TP22, A3, or A20 strains under saline conditions (Table 10). However, applying BacNorte, A3, and A20 bacterial strains increases the chlorophyll B content under saline conditions (Table 11). These results indicate that bacterial strains TP22, A3, and A20 could improve plant growth and alleviate the stress caused by the presence of salts in comparison to the T0 control conditions.

### 2.7. Bioherbicide Activity of the Strains J19, TP22, A20, and A3 Against the Weed Cenchrus Echinatus

**Figure 3:**
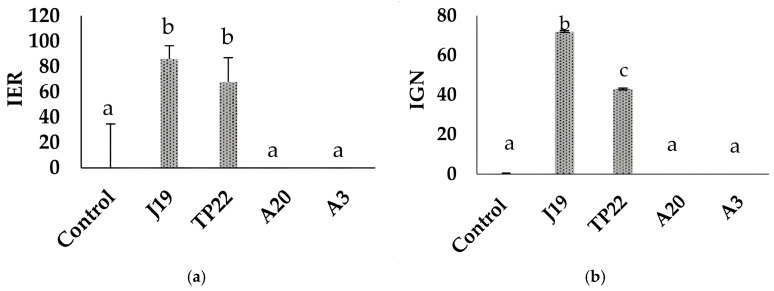
Effect of bacteria as J19, TP22, A3, or A20 strains on *C. echinatus* seeds: (**a**) Normalized residual radical elongation after the experiment; (**b**) Normalized residual percentage of germinated seeds after the experiment. The different letters indicate significant differences from each other (*p* < 0.05; HSD Tukey).

The strains J19, TP22, A20, and A3 were evaluated to determine their effects on the weed *Cenchrus echinatus* (cadillo) growth. Treatments with strains J19 and TP22 showed a significant difference in root elongation compared to the control strains A20 and A3, inhibiting weed growth by 86% for strain J19 and 68% for strain TP22 (Figure 3a).

Significant differences were observed among treatments with the strains J19, TP22, A20, A3, and the control (without inoculation) regarding the inhibitory effect on weed germination. The strain J19 exhibited the highest inhibitory effect, with a germination reduction of 72%, while strain TP22 showed a moderate reduction at 43% (Figure 3b).

## 3. Discussion

Soil salinity has become a significant threat to agricultural ecosystems worldwide, being one of the most important causes of crop productivity reduction [13]. Approximately 1.4 billion hectares of land worldwide are affected by salinity, with 76 million hectares impacted by human-induced salinization [14]. Salt excess negatively impacts the physiological processes in plants, including seed germination, photosynthesis, membrane transport, antioxidants, and ethylene production [15]. Under the soil salinity scenario, plant physiology is not the only one affected. Furthermore, agricultural bioproducts often exhibit inconsistent performance because the microorganisms formulated in these products are naturally adapted to thrive in environments with conditions similar to those from which they were originally sourced [8]. In this sense, the current trend has focused on the search for salt-tolerant microorganisms, which have remarkably succeeded in enhancing the productivity of saline soils [12,13,16].

In this study, four bacterial strains corresponding to isolates J19, TP22, A20, and A3 (Table 1 and Table 2) were selected from the bacterial culture collection of the Laboratory of Plant Pathology and Bioproducts belonging to the Universidad de Tarapacá due to their promising in vitro PGP activities [12]. These bacteria are members of the *Pseudomonas* and *Bacillus* genera, the strain J19 closely related to *P. aeruginosa* (99.93%), the strain TP22 to *B. velezensis* (99.92%), the strain A20 to *P. rhodesiae* (99.80%), and the strain A3 to *B. sanguinis* (99.81%). The four strains were isolated from three geothermal sites, which exhibit high concentrations of salts and boron [12], similar to the cultivation conditions of the Arica and Parinacota Region (Atacama Desert). Based on this information, it is possible to assume that these strains are naturally adapted to saline-boric conditions and are good candidates for developing new bio-based agricultural products for arid environments. Analysis of the tolerance of these strains to NaCl and H_3_BO_3_ (Table 3) showed abundant growth in liquid medium supplemented with both compounds, even when 6.88 g/L NaCl and 912 ppm H_3_BO_3_ (8× Lluta irrigation water) were used. These results agree with Muñoz-Torres et al. [16], who isolated the NaCl and H_3_BO_3_-tolerant *Pseudomonas lini* strain S57 from the oregano roots from Socoroma, Chile. The S57 strain possesses PGP traits and biocontrol activity against phytopathogenic fungi and nematodes. It can grow in a liquid medium supplemented with NaCl and boron in concentrations up to 9× the irrigation water of the Lluta River. Khan et al. [17] studied the effect of the inoculation of the PGP rhizobacterium (PGPR) *Bacillus pumilus* in rice (*Oryza sativa* L.) seedlings maintained in pots and stressed with high concentrations of boron (10 ppm) and NaCl (150 mM). The authors reported that applying *B. pumilus* promoted plant growth under individual stress conditions by increasing the activity of specific antioxidative enzymes. Under combined stress treatments, *B. pumilus* significantly reduced Na⁺ accumulation in rice leaves, although it did not impact leaf boron content. Additionally, the restricted Na⁺ uptake led to reduced antioxidative activity in the plant, regardless of the elevated boron concentrations in the leaves, ultimately enhancing rice tolerance to stress.

Applying salt-tolerant PGP bacteria has proven highly effective in boosting agricultural productivity in saline soils [18,19,20]. PGP bacteria support plant growth through multiple mechanisms, including the production of phytohormones, siderophores, antioxidants, exopolysaccharides (EPS), osmoprotectants, and the enzyme 1-aminocyclopropane-1-carboxylate (ACC) deaminase, along with improved nutrient uptake and induction of systemic resistance under salt stress [13]. Different PGP activities were detected for the J19, TP22, A20, and A3 strains (Table 4). For example, the four isolates commonly contained hydrolytic enzymes (proteases, lipases, cellulases, and chitinases) involved in the degradation of organic matter. Soil organic matter comprises materials generated by various organisms that return to the soil and undergo decomposition. It plays a crucial role in determining soil quality and fertility. This decomposition process is mediated by extracellular enzymes, such as microbial hydrolases, secreted into the environment to break down macromolecules into soluble substrates, facilitating their assimilation [21]. Hydrolases from the strains J19, TP22, A20, and A3 would participate in the decomposition of organic matter, making it available for the plant and, in this sense, favoring its growth.

Moreover, the four strains had different PGP traits, including the production of auxins, HCN, and siderophores, phosphate solubilization, nitrogen fixation, and production of ACC deaminase. Furthermore, biocontrol activity against phytopathogenic fungi was detected in the strains J19, TP22, and A3 (Table 4). The presence of PGP traits in bacteria isolated from geothermal sites has been studied previously by Patel et al. [22]. The authors isolated 123 bacterial strains from natural hot springs located in Unnai and Tuwa (India) and screened them to evaluate the PGP properties of these isolates. Their analysis showed the presence of antagonistic activity against different phytopathogens, the production of auxins, the solubilization of phosphate, and the production of hydrolytic enzymes (lipase, protease, and cellulase). Half of the isolated showed 5% NaCl (*w*/*v*) tolerance, indicating that these microorganisms may affect crop production in saline and arid environments. Recently, Sood et al. [23] isolated nineteen bacterial strains from Tattapani and Sohna Hot Springs. 16S rDNA sequencing of the selected strains revealed a predominance of the families Bacillaceae, Pseudomonadaceae, Moraxellaceae, Enterobacteriaceae, Paracoccaceae, and Acidithiobacillaceae. Most bacterial strains exhibited antagonistic activity and could solubilize zinc, potassium, phosphate, and silicate under in vitro conditions. Among these strains, 84% showed siderophore production, while 73% demonstrated indole-3-acetic acid (IAA) production. These studies indicate the potential for isolating extremotolerant bacteria from extreme habitats to develop new bio-based products applicable to agriculture across diverse environments, with production under arid and semiarid conditions of particular interest.

PGP bacteria are known to increase plant growth and enhance plant salt tolerance [24,25]. It was of particular interest in this study that the strains A20 and A3 stimulate the growth of tomato plants cv. Poncho Negro under saline conditions, specifically in the aerial part of the plant (Table 7 and Table 8). However, no growth stimulation was observed without NaCl (Table 7 and Table 8). This phenomenon could be explained by the source of these microorganisms’ isolation. As observed, these bacteria are naturally adapted to growth under saline environments (Table 1 and Table 3), allowing their cellular machinery to function optimally under saline conditions. In this sense, the presence of NaCl would stimulate bacterial growth and activate the activity of different enzymes involved in the production of secondary metabolites (such as siderophores and phytohormones) and in plant growth regulation, such as ACC deaminase, which is present in the strain A20.

Moreover, the inoculation of the bacterial strains TP22, A20, and A3 significantly increases the proline content (Table 9) and the chlorophyll A concentration (Table 10) in tomato plants cv. Poncho Negro under saline conditions, similar to the observed when these plants were inoculated with the commercial biostimulant BacNorte. Similarly, applying strains A20, A3, and BacNorte increased the chlorophyll B content in the presence of 0.1 M NaCl compared to the T0 treatment (Table 11). Prittesh et al. [26] isolated nine salt-tolerant bacteria with PGP activity belonging to the *Bacillus*, *Exiguobacterium*, *Enterobacter*, *Lysinibacillus*, *Stenotrophomonas*, *Microbacterium*, and *Achromobacter* genera. The authors determined that bacterial strains’ inoculation stimulates rice plants’ growth under saline stress.

Furthermore, bacterial inoculants significantly increased total chlorophyll, proline, and total phenol levels while mitigating oxidative damage indicators such as electrolyte leakage and improving the membrane stability index under salt stress. These findings suggest that salt-tolerant PGP bacteria could effectively promote the growth of *O. sativa* in salinized agricultural soils. Other studies have found that inoculating PGPB increases the chlorophyll and proline content in cucumbers grown under saline stress [27].

Salinity stress has been reported to reduce chlorophyll synthesis by downregulating photosynthesis [28], probably due to increased Na⁺ concentrations. However, inoculation with salt-tolerant strains significantly increased photosynthetic pigment levels. This pigment recovery in inoculated plants indicates that PGPBs promote induced systemic tolerance under NaCl stress. Similar findings have been observed in studies where inoculation with *Paenibacillus yonginensis*, *Sinorhizobium meliloti*, *Bacillus aryabhattai*, and *Bacillus mesonae* enhanced chlorophyll content in *Medicago sativa*, *Panax ginseng*, and *Solanum lycopersicum* under salinity stress [28,29,30]. Additionally, proline accumulation under stress conditions has been linked to enhanced stress tolerance, showing a positive correlation between proline levels in plant cells and salt tolerance [31,32]. Beyond this correlation, proline plays a critical role in osmotic regulation and acts as a free radical scavenger within plant cells [31,33]. The results of this work suggest that strains A20 and A3 not only stimulate tomato plant growth but also mitigate the effect of saline stress through the accumulation of proline and the increase in chlorophyll content.

Regarding bacteria’s in vitro biocontrol activity against the weed *C. echinatus*, strain J19 inhibited germination by 72% and root elongation by 86%. In comparison, strain TP22 inhibited germination by 43% and root elongation by 68% in *C. echinatus* (Figure 3). It has been reported that the bacterium *P. aeruginosa* strain A52 exhibits strong herbicidal potential, inhibiting the growth of weeds such as *Axonopus affinis* and *Lens esculenta* by 75% and 50%, respectively [34]. Additionally, *P. aeruginosa* completely inhibited germination, root growth, and shoot development in dicotyledonous weed seeds, with invasive weeds showing higher sensitivity and lower effectiveness observed in monocotyledonous weeds; the seeds that managed to germinate displayed stunted growth due to the presence of quinoline and exposure to high levels of HCN [35,36]. Regarding phytotoxic effects, strain J19 demonstrated high toxicity in the germination index (IGN), while strain TP22 exhibited moderate toxicity. Root elongation is considered more critical than germination due to its physiological processes, as toxic substances can disrupt normal plant development. However, these volatile-producing bacteria do not necessarily eradicate weeds but could suppress initial weed growth, thereby facilitating the development of cultivated plants impacted by weeds [36].

Biocontrol agents and biostimulants are susceptible to environmental factors such as temperature, moisture, and salinity, making their adaptation to new environments a significant challenge. Many agents that perform effectively under controlled laboratory conditions often fail to replicate such success in field applications. Despite their potential, only a limited number of bioproducts have been successfully commercialized. While microbial-based products are in use and show promise, the process of registering and commercializing these agents remains complex, limiting their widespread adoption. Developing appropriate formulations is critical to introducing and establishing bioproducts in new ecosystems. Formulations must support the bioproduct’s survival, proliferation, and activity in the target environment. Once an agent is selected based on its efficacy and biomass production, the next step involves creating a formulation that is stable, easy to handle, and capable of being effectively delivered to the site of action. These formulations play a vital role in ensuring the bioproduct’s success by enhancing the bioformulation’s adaptability and functionality in field conditions [37].

For successful bioproduct development, generating a highly concentrated microbial culture is essential. Key factors influencing bacterial cell biomass during production include methods that save time and reduce costs. Optimizing growth conditions, such as pH, temperature, agitation, and incubation time, can significantly enhance bacterial biomass yields [38]. For the four strains, pH, temperature, and agitation were modified in flasks to determine optimal parameters for inoculum preparation. Bacterial growth curves were determined by measuring OD_600_ under optimal and non-optimal conditions (Figure 2). Under optimal conditions, strain J19 showed an increase in growth after 4 h of incubation, reaching the stationary phase 10 h post-inoculation (30 °C, 200 rpm, pH 5.5). For strain TP22, the exponential phase began after 4 h, with the stationary phase reaching 11 h (35 °C, 200 rpm, pH 7.5). Strain A20 entered the exponential phase after 7 h, reaching the stationary phase at 12 h under optimal conditions (35 °C, 200 rpm, pH 5.5). Strain A3 exhibited exponential growth at 3 h, reaching the stationary phase at 9 h under optimal conditions of 35 °C, 200 rpm, and pH 7.0. These results represent an increment in biomass production compared to non-optimal conditions and settle the basis for the scale-up of these microorganisms in a bioreactor.

## 4. Materials and Methods

### 4.1. Bacterial Strains and Growth

Four bacterial strains (J19, TP22, A20, and A3) were selected from the bacterial culture collection of the Laboratory of Plant Pathology and Bioproducts at Universidad de Tarapacá for their promising in vitro plant growth-promoting (PGP) activities [13]. The strain J19 was isolated from the Jurasi Hot Springs (18.2104° S, 69.5107° W; temperature range: 32–64 °C; pH: 7.76). The strain TP22 was obtained from the Polloquere Hot Springs (18.9128° S, 68.9987° W; temperature range: 40–75 °C; pH: 6.35). Meanwhile, the strains A20 and A3 were isolated from Amuyo Lagoons (19.0581° S, 69.2528° W; temperature range: 21–52 °C; pH range: 7.01–8.21).

Bacteria were grown in King’s medium B [39] containing the following (per liter): 20.0 g peptone, 10.0 mL glycerol, 1.5 g K_2_HPO_4_, and 1.5 g MgSO_4_·7H_2_O (pH 7.0); and incubated at 25 °C for 1 week.

### 4.2. Bacterial Identification

Genomic DNA from the selected bacterial strains was extracted using the DNeasy UltraClean Microbial Kit (QIAGEN, Germantown, MD, USA), following the manufacturer’s protocol. The 16S rRNA gene was amplified via PCR using bacteria-specific primers 27F (5′-AGAGTTTGATCCTGGCTCAG-3′) and 1492R (5′-CTACGGCTACCTTGTTACGA-3′) [40]. PCR reaction mix and cycling conditions were conducted as outlined by Muñoz et al. [6]. The reaction mixture was composed of 1.0 U of Taq DNA polymerase, 200 μM of each deoxynucleotide triphosphate (dATP, dCTP, dGTP, and dTTP), 1× reaction buffer, 0.75 mM MgCl_2_, and 0.5 mM of each primer. The PCR protocol included an initial denaturation step at 95 °C for 2 min, followed by 40 cycles of denaturation at 95 °C for 45 s, annealing at 55 °C for 45 s, and extension at 72 °C for 45 s. A final extension at 72 °C for 10 min completed the reaction. Amplification reactions were performed using a Veriti™ 96-well thermal cycler (Thermo Fisher Scientific, Waltham, MA, USA). A ~1500 bp band was observed on a 1.0% (*w*/*v*) agarose gel prepared in 1× TAE buffer (40 mM Tris-acetate, 10 mM EDTA) and visualized under UV light using 1× GelRed (Biotium, San Francisco, CA, USA).

PCR products were sequenced using the primers described above by Macrogen (Seoul, Republic of Korea) and manually edited in ChromasPro software v2.1.10.1 (http://technelysium.com.au/wp/chromaspro/ accessed on 1 April 2022) to remove low-quality bases. Forward and reverse sequences were assembled using the Megamerger tool (http://www.bioinformatics.nl/cgi-bin/emboss/megamerger accessed on 1 April 2022) to obtain a final sequence length of ~1500 bp. The partial sequence was compared to entries in GenBank using BLAST software (https://blast.ncbi.nlm.nih.gov/Blast.cgi accessed on 22 November 2024) [41].

### 4.3. Bacterial Characterization

The phenotypic characterization of the strains J19, TP22, A20, and A3 was conducted following the methodology outlined by Muñoz et al. [42].

Antibiotic susceptibility testing was performed using the disk diffusion method described by Simirgiotis et al. [43], employing different antibiotics, as shown in Table 2. The quantity of each antibiotic was 25 μg for amoxicillin and ampicillin, 50 μg for chloramphenicol, 10 μg for ciprofloxacin, 30 μg for kanamycin and neomycin, and 10 U for penicillin G. The assay included five independent replicates to ensure reliability.

### 4.4. Tolerance of the Bacterial Strains to NaCl and H_3_BO_3_

The NaCl tolerance of the strains J19, TP22, A20, and A3 was assessed using King’s medium B supplemented with NaCl concentrations ranging from 0 to 30 g/L. H_3_BO_3_ tolerance was similarly evaluated in King’s medium B amended with 0–300 ppm of H_3_BO_3_. A modified King’s medium B containing 0.86 g/L NaCl and 114 ppm H_3_BO_3_ (1×) was used to simulate the composition of irrigation water from the Lluta River, which is the primary source of water for crops in the Lluta Valley [44]. This setup allowed testing of bacterial growth under conditions that mimic the extreme water characteristics of the region. Concentrations were also tested at higher values, up to 8 times the irrigation water of the Lluta River.

After one day of incubation at 25 °C, bacterial growth was monitored by measuring OD_600_ with a spectrophotometer. All tests were conducted in triplicate to ensure accuracy.

### 4.5. In Vitro PGP Traits

Protease activity was assessed by culturing the bacteria on skim milk agar medium prepared with 28.0 g skim milk, 5.0 g casein hydrolysate, 2.5 g yeast extract, 1.0 g glucose, and 15.0 g agar per liter. The cultures were incubated at room temperature for five days. Proteolytic activity was indicated by a clear halo surrounding the bacterial colony on a white background, signifying protein degradation [45].

The cellulolytic activity was assessed on agar plates containing 1.0 g carboxymethylcellulose, 1.0 g peptone, 0.3 g urea, 2.0 g KH_2_PO_4_, 1.0 g (NH_4_)_2_SO_4_, 0.3 g CaCl_2_, 0.3 g/L MgSO_4_, 0.014 g/L ZnSO_4_, 0.002 g/L CoCl_2_, 0.05 g/L FeSO_4_, 0.016 g/L MnSO_4_, and 15.0 g agar per liter. The cultures were incubated at room temperature for five days, after which the plates were treated with 0.1% Congo red solution for 20 min, followed by two washes with 1 M NaCl for 20 min each. The cellulolytic activity was indicated by a transparent halo surrounding the bacterial colony against a red background [46].

Lipase activity was assessed following the method outlined by Slifkin [47]. The Tween 80 agar medium was prepared with 10.0 g peptone, 5.0 g NaCl, 0.1 g CaCl_2_, 5.0 mL Tween 80, and 15.0 g agar per liter. The cultures were incubated at room temperature for five days. A white halo around the microorganism indicated a positive result for lipase activity, signaling the hydrolysis of Tween 80. This reaction produces fatty acids interacting with calcium ions in the medium, forming a white precipitate.

Chitinase activity was determined using colloidal chitin agar as described by Verma and Garg [48]. The medium composition per liter included 20.0 g colloidal shrimp chitin, 0.5 g yeast extract, 1.0 g MgSO_4_·7H_2_O, 1.36 g KH_2_PO_4_, and 15.0 g agar. The cultures were incubated at room temperature for five days. After incubation, chitinase activity was indicated by a clear halo surrounding the bacterial colony on a white background, signifying chitin degradation.

Indole-3-acetic acid (IAA) production was determined using the colorimetric Salkowski method [49]. After centrifuging the bacterial culture (4000 rpm for 10 min), the resulting supernatant was mixed with Salkowski’s reagent (0.5 M FeCl_3_ in 35% HClO_4_) in a 1:1 ratio. A color shift from yellow to red confirmed the presence of IAA.

Siderophore production was evaluated using the Chrome Azurol S (CAS) assay following the method of Schwynand and Neilands [50]. This procedure combined 500 μL of bacterial supernatant (obtained by centrifugation at 4000 rpm for 10 min) with an equal volume of CAS solution. A color change from blue to orange signified siderophore production.

The solubilization of inorganic phosphate was assessed on Pikovskaya (PVK) solid medium [51]. The PVK medium composition per liter contained 10.0 g glucose, 0.5 g yeast extract, 0.5 g (NH_4_)_2_SO_4_, 0.1 g MgSO_4_·7H_2_O, 5.0 g Ca_3_(PO_4_)_2_, 0.2 g KCl, 0.002 g MnSO_4_·2H_2_O, 0.002 g FeSO_4_·7H_2_O, and 15.0 g agar. The cultures were incubated at room temperature for five days. A clearing zone around bacterial colonies after incubation indicated phosphate solubilization activity.

The nitrogen-fixing capability of each bacterial isolate was evaluated using NFb semisolid medium [52]. This medium was composed of DL-malic acid (5.0 g/L), K_2_HPO_4_ (0.5 g/L), MgSO_4_·7H_2_O (0.2 g/L), NaCl (0.1 g/L), CaCl_2_·2H_2_O (0.02 g/L), agar (0.5 g/L), a micronutrient solution (2 mL/L), bromothymol blue solution (2 mL/L of 0.5% in 0.2 N KOH), Fe (III)-EDTA (4 mL/L of 1.64% *w*/*v*), and a vitamin solution (1 mL/L). The pH was adjusted to 6.8 with NaOH. The micronutrient solution contained CuSO_4_·5H_2_O (0.4 g/L), ZnSO_4_·7H_2_O (0.12 g/L), H_3_BO_3_ (1.4 g/L), Na_2_MoO_4_·2H_2_O (1.0 g/L), and MnSO_4_·H_2_O (1.5 g/L). The vitamin solution comprised biotin (100 mg/L) and pyridoxal HCl (200 mg/L). Nitrogen fixation was indicated by forming a sub-surface whitish “veil-like” pellicle following incubation.

ACC deaminase activity was assessed by cultivating the bacterial isolates in a medium where 3 mM of 1-aminocyclopropane-1-carboxylic acid served as the sole nitrogen source, following the methodology established by Penrose and Glick [53].

The production of HCN was determined by using strips of filter paper moistened with a picrate solution (0.5% picric acid in a 2% sodium carbonate solution) fixed on the lid of the Petri dish. The dish contained the culture in King’s B medium supplemented with 4.4 g of glycine and incubated at 28 °C. The change in the paper’s color from yellow to orange-brown within 48 h indicated HCN production [54].

An antagonism assay was conducted using 94 mm Petri plates with 20 mL of PDA, following the procedure outlined by Sepúlveda-Chavera [55]. Fresh PDA plates were inoculated with agar disks from one-week-old, actively growing colonies of phytopathogenic fungi, including *Botrytis cinerea*, *Monilinia fructicola*, *Geotrichum candidum*, *Fusarium oxysporum*, and *Macrophomina phaseolina*, sourced from the Microbial Culture Collection of the Universidad de Tarapacá, placed in the center of the plates. In parallel, 20 μL of each bacterial isolate was inoculated in 5 mm wells positioned 2.5 cm away from the center. Plates with only fungal inoculum served as controls. The cultures were incubated at room temperature for 5–7 days, and a halo around the bacterial wells indicated fungal growth inhibition.

All assays were performed in triplicate.

### 4.6. Bacterial Culture Optimization and Optimal Bacterial Growth Curve

A growth curve was generated by taking 1.5 mL aliquots of culture medium inoculated with the selected bacterium. Bacterial growth was evaluated by measuring the increase in OD_600_ using a spectrophotometer, and serial dilutions monitored viable counts on a solid medium. This process involved independently modifying the agitation (0–200 rpm), temperature (25–40 °C), and pH (5.0–8.0); for the determination of optimum temperature, pH and agitation were fixed at 7.0 and 100 rpm, respectively. The temperature and agitation were fixed at 30 °C and 100 rpm for optimum pH. The temperature and pH were set at 30 °C and pH 7.0 to determine the optimum agitation.

Flask experiments were conducted until the stationary phase using a final volume of 200 mL of bacterial culture. The effect of these parameters was analyzed by generating bacterial growth curves and obtaining the microbial growth rate (µ) and generation time (g). Generation time was determined using the equation: g = Log 2/m, where m is the slope of the line derived from absorbance values over incubation time during the exponential growth phase. The growth rate (µ) was determined with the equation µ = 1/g, where g is the generational time, to obtain optimal parameters.

The optimal bacterial growth curve was compared with the growth under non-optimal conditions (room temperature, no agitation, and neutral pH). Flask experiments were monitored until the stationary phase by measuring OD_600_ with a spectrophotometer, using a final volume of 200 mL of bacterial culture, and taking 1.5 mL aliquots of culture medium inoculated with selected bacterium.

### 4.7. PGP Activity of the Strains J19, TP22, A20, and A3 in Tomato Plants cv. Poncho Negro

PGP activity was also assessed for tomato plants cv. Poncho Negro. Tomato seeds were disinfected by immersion in 95% (*v*/*v*) ethanol for 2 min, followed by 2% (*v*/*v*) sodium hypochlorite for 2 min, and a final wash in 70% (*v*/*v*) ethanol for 2 min. The seeds were then rinsed twice with sterile distilled water for 2 min each. Seeds were germinated in a sterile, dark, wet chamber at 25 °C for seven days. Following germination, the seedlings were transferred to pots filled with twice-autoclaved vermiculite as the sole substrate and placed in a greenhouse. The plants were treated once a week for a month with 1 × 10^8^ CFU of each strain applied at the base of the stem. The inoculum was freshly prepared and diluted in sterile water. Control plants (T0) were treated with sterile King’s medium B.

A positive control based on the commercial product BacNorte (halotolerant *B. velezensis*) was also used to compare stimulation results. The plants were watered with tap water. After four weeks of inoculation, the tomato plants were removed from the substrate and washed, and measurements were taken for stem and root lengths and the wet and dry weights of the roots and aerial parts. The same experiment was conducted using a 0.1 M NaCl solution instead of tap water. All experiments were conducted with five independent replicates.

Proline concentration in leaves was determined by the method described by Ábrahám et al. [56]. Chlorophyll A and B were measured according to the methodology described by Sinha et al. [57].

### 4.8. Herbicidal Activity of the Strains J19, TP22, A20 and A3 Against the Weed Cenchrus Echinatus

An in vitro assay was conducted to test the germination inhibition of the weed *C. echinatus* (cadillo) in wet chambers. Weed seeds were disinfected with 95% ethanol for 2 min, 2% sodium hypochlorite for 5 min, and 70% ethanol for 2 min, followed by three rinses with sterilized distilled water. After disinfection, the seeds were sown on Petri dishes containing PDA medium and incubated for 48 h. After this incubation, the bases of the Petri dishes containing the weed seeds on PDA medium were paired with a Petri dish containing bacterial inoculum of strains, which had been previously streaked on King’s medium B supplemented with glycine. The plates were then sealed with Parafilm and incubated at 28 °C for an additional 92 h.

The bacteria ‘s effect on the weed was determined using the normalized residual germination index (IGN) and the normalized residual root elongation index (IER) [34].

The IGN was determined using the equation:

$$IGN=\frac{\left({Germ}_{x}-{Germ}_{Control}\right)}{{Germ}_{control}}$$

where Germ_x_ represents the average germination percentage of seeds in bacterial inoculum, and Germ_control_ represents the germination percentage of seeds without inoculation.

The IER was determined using the equation:

$$IER=\frac{\left({Elong}_{x}-{Elong}_{Testigo}\right)}{{Elong}_{Testigo}}$$

where Elong_x_ represents the average root length of seeds germinating with bacterial inoculum, while Elong_control_ represents the average root length of seeds germinating without inoculation.

## 5. Conclusions

This study determined the effect of four bacterial strains with different PGP activities isolated from three geothermal sites. The strains belong to the *Pseudomonas* (J19 and A20) and *Bacillus* (TP22 and A3) genera and can tolerate saline-boric conditions, such as those present in the Arica and Parinacota Region (Atacama Desert). Strains A3 and A20 could improve plant growth and survival under saline stress by establishing beneficial interactions with tomato plants grown under saline conditions. Meanwhile, the strains J19 and TP22 have herbicidal activity against the weed *C. echinatus*. These bacterial strains demonstrate plant growth-promoting traits and the potential to improve plant resistance to salinity and boron stress. Their characteristics position them as valuable candidates for developing innovative bio-based agricultural products tailored for arid and semi-arid regions.

## 6. Patents

A patent application was requested to INAPI under the application code PCT/CL2022/050102.

## Figures and Tables

**Table 1:** Microbiological characterization of strains J19, TP22, A20 and A3. A positive reaction is indicated as +; meanwhile, no reaction is indicated as -.

Parameter	J19	TP22	A20	A3
Source	Jurasi Hot Springs	Polloquere Hot Springs	Amuyo Lagoons	Amuyo Lagoons
Genus	*Pseudomonas*	*Bacillus*	*Pseudomonas*	*Bacillus*
Affiliation	*Pseudomonas aeruginosa* (NR_117678.1)	*Bacillus velezensis* (NR_075005.2)	*Pseudomonas rhodesiae * (NR_024911.1)	*Bacillus sanguinis* (NR_175555.1)
Similarity (%)	99.93	99.92	99.80	99.81
Gram staining	-	+	-	+
Morphology	Straight rods	Straight rods	Straight rods	Straight rods
Motility test	+	+	-	+
Acid production from:				
L-Asparagine	-	-	-	-
D-Glucose	+	+	+	-
D-Galactose	+	-	+	-
D-Cellobiose	-	+	+	+
D-Melibiose	-	-	+	-
D-Fructose	-	-	+	+
D-Rhamnose	-	-	+	-
D-Raffinose	-	+	-	-
D-Mannitol	-	+	+	-
D-Sorbitol	-	+	-	-
D-Inositol	-	-	-	-
Sucrose	-	+	-	-
Lactose	-	+	-	-

**Table 2:** Antibiotic susceptibility test of strains J19, TP22, A20, and A3. The sensitive susceptibility is represented as S; intermediate susceptibility as I and resistant susceptibility as R.

Antibiotic	Quantity	Susceptibility			
		J19	TP22	A20	A3
Amoxicillin	25 µg	R	R	R	R
Ampicillin	25 µg	R	R	R	R
Chloramphenicol	50 µg	I	S	S	S
Ciprofloxacin	10 µg	S	S	S	I
Kanamycin	30 µg	R	S	S	S
Neomycin	30 µg	R	S	S	I
Penicillin G	10 U	R	R	R	R

Sensitive (>20 mm), intermediate susceptibility (15 mm–19 mm), resistance (<14 mm) according to Clinical and Laboratory Standards Institute (2020).

**Table 3:** Tolerance of strains J19, TP22, A20, and A3 to NaCl and H_3_BO_3_. Error corresponds to the standard deviation of three independent assays. The different letters indicate significant differences from each other (*p* < 0.05; HSD Tukey).

Condition	Bacterial Growth			
	J19	TP22	A20	A3
Control	0.76 ± 0.14 ^a^	1.23 ± 0.43 ^a^	0.68 ± 0.22 ^a^	0.21 ± 0.10 ^a^
8 g/L NaCl	0.76 ± 0.05 ^a^	1.02 ± 0.03 ^a^	0.50 ± 0.03 ^a^	0.16 ± 0.02 ^a^
15 g/L NaCl	0.68 ± 0.08 ^a^	0.98 ± 0.05 ^a^	0.54 ± 0.01 ^a^	0.15 ± 0.06 ^a^
30 g/L NaCl	0.48 ± 0.06 ^b^	0.42 ± 0.01 ^b^	0.54 ± 0.02 ^a^	0.17 ± 0.02 ^a^
10 ppm H_3_BO_3_	0.62 ± 0.06 ^a^	0.86 ± 0.04 ^a^	0.49 ± 0.01 ^a^	0.10 ± 0.02 ^a^
100 ppm H_3_BO_3_	0.56 ± 0.01 ^b^	0.83 ± 0.05 ^a^	0.44 ± 0.04 ^b^	0.08 ± 0.01 ^a^
300 ppm H_3_BO_3_	0.58 ± 0.03 ^b^	0.81 ± 0.02 ^a^	0.38 ± 0.00 ^b^	0.10 ± 0.03 ^a^
1× Lluta irrigation water ^1^	0.83 ± 0.22 ^a^	1.52 ± 0.14 ^a^	1.13 ± 0.27 ^a^	0.22 ± 0.02 ^a^
2× Lluta irrigation water ^1^	0.89 ± 0.09 ^a^	1.55 ± 0.11 ^a^	1.10 ± 0.10 ^a^	0.29 ± 0.02 ^a^
4× Lluta irrigation water ^1^	0.81 ± 0.09 ^a^	1.50 ± 0.16 ^a^	0.96 ± 0.16 ^a^	0.27 ± 0.01 ^a^
8× Lluta irrigation water ^1^	0.75 ± 0.02 ^a^	1.47 ± 0.01 ^a^	0.41 ± 0.01 ^b^	0.32 ± 0.01 ^a^

^1^ The liquid medium was supplemented with concentration 0.86 g/L NaCl and 114 ppm H_3_BO_3_ (1× Lluta irrigation water).

**Table 4:** In vitro PGP traits of strains J19, TP22, A20, and A3. A positive reaction is indicated as +; meanwhile, no reaction is indicated as -.

Characteristic	J19	TP22	A3	A20
Protease production	+	+	+	+
Cellulase production	+	-	+	+
Lipase production	+	-	+	+
Chitinase production	-	+	-	-
Auxin production	+	-	+	+
Siderophores production	+	-	-	-
Phosphate solubilization	+	-	+	+
Nitrogen fixation	+	+	-	+
ACC deaminase production	+	-	-	+
HCN production	+	-	-	-
Biocontrol against *Botrytis cinerea*	+	+	+	-
Biocontrol against *Fusarium oxysporum*	+	+	-	-
Biocontrol against *Geotrichum candidum*	+	+	+	-
Biocontrol against *Macrophomina phaseolina*	+	+	+	-
Biocontrol against *Monilinia fructicola*	+	+	+	-

**Table 5:** Effect of the inoculation of the strain J19 on the growth of Poncho Negro tomato plants treated with 0 M and 0.1 M of NaCl. Error represents the standard deviation of the five independent assays. The different letters indicate significant differences from each other (*p* < 0.05; HSD Tukey). Letters should be compared within the column. T0 corresponds to non-inoculated tomato plants.

Treatment	Root Length (cm)	Root Fresh Weight (g)	Root Dry Weight (g)	Aerial Length (cm)	Aerial Fresh Weight (g)	Aerial Dry Weight (g)
T0	40.07 ± 5.71 ^a^	6.92 ± 0.72 ^a^	0.68 ± 0.14 ^a^	32.82 ± 3.85 ^a^	23.13 ± 5.17 ^a^	2.95 ± 0.61 ^a^
BacNorte	38.94 ± 4.45 ^a^	3.91 ± 0.74 ^b^	0.58 ± 0.09 ^a^	24.90 ± 5.81 ^b^	20.96 ± 4.71 ^a^	2.46 ± 0.42 ^a^
J19	41.44 ± 2.90 ^a^	4.80 ± 0.82 ^b^	0.64 ± 0.17 ^a^	31.62 ± 3.76 ^ab^	20.20 ± 4.38 ^a^	2.57 ± 0.49 ^a^
T0 + 0.1 M NaCl	45.80 ± 4.95 ^a^	3.91 ± 1.27 ^ab^	0.58 ± 0.17 ^a^	23.69 ± 1.40 ^a^	17.49 ± 2.84 ^a^	2.24 ± 0.35 ^a^
BacNorte + 0.1 M NaCl	40.30 ± 5.72 ^a^	2.95 ± 1.16 ^a^	0.61 ± 0.09 ^a^	27.70 ± 2.08 ^b^	20.20 ± 2.86 ^a^	2.72 ± 0.21 ^a^
J19 + 0.1 M NaCl	43.70 ± 5.22 ^a^	4.79 ± 0.51 ^b^	0.50 ± 0.09 ^a^	30.10 ± 2.56 ^b^	21.23 ± 4.22 ^a^	2.63 ± 0.45 ^a^

**Table 6:** Effect of the inoculation of the strain TP22 on the growth of Poncho Negro tomato plants treated with 0 M and 0.1 M of NaCl. Error represents the standard deviation of the five independent assays. The different letters indicate significant differences from each other (*p* < 0.05; HSD Tukey). Letters should be compared within the column. T0 corresponds to non-inoculated tomato plants.

Treatment	Root Length (cm)	Root Fresh Weight (g)	Root Dry Weight (g)	Aerial Length (cm)	Aerial Fresh Weight (g)	Aerial Dry Weight (g)
T0	40.07 ± 5.71 ^a^	6.92 ± 0.72 ^a^	0.68 ± 0.14 ^a^	32.82 ± 3.85 ^a^	23.13 ± 5.16 ^a^	2.95 ± 0.61 ^a^
BacNorte	38.94 ± 4.45 ^a^	3.91 ± 0.74 ^b^	0.58 ± 0.09 ^a^	24.90 ± 5.81 ^a^	20.96 ± 4.71 ^a^	2.46 ± 0.42 ^a^
TP22	40.76 ± 8.65 ^a^	5.23 ± 1.80 ^ab^	0.73 ± 0.22 ^a^	32.14 ± 4.55 ^a^	23.20 ± 7.44 ^a^	2.99 ± 1.05 ^a^
T0 + 0.1 M NaCl	45.80 ± 4.95 ^a^	3.91 ± 1.27 ^a^	0.58 ± 0.17 ^a^	23.69 ± 1.40 ^a^	17.49 ± 2.84 ^a^	2.24 ± 0.35 ^a^
BacNorte + 0.1 M NaCl	40.30 ± 5.72 ^a^	2.95 ± 1.16 ^a^	0.61 ± 0.09 ^a^	27.70 ± 2.08 ^a^	20.20 ± 2.86 ^ab^	2.72 ± 0.21 ^ab^
TP22 + 0.1 M NaCl	49.20 ± 9.38 ^a^	6.76 ± 1.61 ^b^	0.71 ± 0.15 ^a^	31.30 ± 8.29 ^a^	29.65 ± 9.33 ^b^	3.74 ± 1.16 ^b^

**Table 7:** Effect of the inoculation of the strain A20 on the growth of Poncho Negro tomato plants treated with 0 M and 0.1 M of NaCl. Error represents the standard deviation of the five independent assays. The different letters indicate significant differences from each other (*p* < 0.05; HSD Tukey). Letters should be compared within the column. T0 corresponds to non-inoculated tomato plants.

Treatment	Root Length (cm)	Root Fresh Weight (g)	Root Dry Weight (g)	Aerial Length (cm)	Aerial Fresh Weight (g)	Aerial Dry Weight (g)
T0	40.07 ± 4.95 ^a^	6.92 ± 0.72 ^a^	0.68 ± 0.14 ^a^	32.82 ± 3.85 ^a^	23.13 ± 5.16 ^a^	2.95 ± 0.61 ^a^
BacNorte	38.94 ± 5.72 ^a^	3.91 ± 0.74 ^b^	0.58 ± 0.09 ^a^	24.90 ± 5.81 ^b^	20.96 ± 4.71 ^a^	2.46 ± 0.43 ^a^
A20	39.20 ± 1.52 ^a^	4.56 ± 2.05 ^b^	0.76 ± 0.24 ^a^	33.92 ± 2.61 ^a^	27.20 ± 7.10 ^a^	3.52 ± 0.96 ^a^
T0 + 0.1 M NaCl	45.80 ± 4.95 ^a^	3.91 ± 1.26 ^a^	0.58 ± 0.17 ^a^	23.69 ± 1.4 ^a^	17.49 ± 2.84 ^a^	2.24 ± 0.35 ^a^
BacNorte + 0.1 M NaCl	40.03 ± 5.72 ^a^	2.95 ± 1.16 ^a^	0.61 ± 0.09 ^a^	27.70 ± 2.08 ^b^	20.20 ± 2.86 ^a^	2.72 ± 0.21 ^a^
A20 + 0.1 M NaCl	46.90 ± 1.52 ^a^	7.52 ± 1.59 ^b^	0.70 ± 0.13 ^a^	33.10 ± 2.07 ^c^	30.05 ± 3.76 ^b^	3.55 ± 0.39 ^b^

**Table 8:** Effect of the inoculation of strain A3 on the growth of Poncho Negro tomato plants treated with 0 M and 0.1 M of NaCl. Error represents the standard deviation of the five independent assays. The different letters indicate significant differences from each other (*p* < 0.05; HSD Tukey). Letters should be compared within the column. T0 corresponds to non-inoculated tomato plants.

Treatment	Root Length (cm)	Root Fresh Weight (g)	Root Dry Weight (g)	Aerial Length (cm)	Aerial Fresh Weight (g)	Aerial Dry Weight (g)
T0	40.07 ± 5.71 ^a^	6.92 ± 0.72 ^a^	0.68 ± 0.14 ^a^	32.82 ± 3.85 ^a^	23.13 ± 5.16 ^a^	2.95 ± 0.61 ^a^
BacNorte	38.94 ± 4.45 ^a^	3.91 ± 0.74 ^b^	0.58 ± 0.09 ^a^	24.90 ± 5.81 ^b^	20.96 ± 4.71 ^a^	2.46 ± 0.42 ^a^
A3	45.18 ± 9.75 ^a^	4.84 ± 1.37 ^b^	0.76 ± 0.21 ^a^	33.90 ± 2.47 ^a^	23.61 ± 4.84 ^a^	2.96 ± 0.69 ^a^
T0 + 0.1 M NaCl	45.80 ± 4.95 ^a^	3.91 ± 1.27 ^ab^	0.58 ± 0.17 ^a^	23.69 ± 1.40 ^a^	17.49 ± 2.84 ^a^	2.24 ± 0.35 ^a^
BacNorte + 0.1 M NaCl	40.30 ± 5.72 ^a^	2.95 ± 1.16 ^a^	0.61 ± 0.09 ^a^	27.70 ± 2.08 ^a^	20.20 ± 2.86 ^a^	2.72 ± 0.21 ^a^
A3 + 0.1 M NaCl	47.60 ± 6.74 ^a^	5.59 ± 1.94 ^b^	0.81 ± 0.22 ^a^	33.46 ± 4.69 ^b^	31.84 ± 9.63 ^b^	4.05 ± 1.22 ^b^

**Table 9:** Effect of the inoculation of the bacterial strain J19, TP22, A20, and A3 on the proline content of Poncho Negro tomato plants under standard and saline conditions. Error represents the standard deviation of the five independent assays. The different letters indicate significant differences from each other (*p* < 0.05; HSD Tukey). T0 corresponds to non-inoculated tomato plants.

Treatment	[Proline] (μg/mL)
T0	6.99 ± 1.59 ^a^
BacNorte	6.45 ± 0.85 ^a^
J19	7.74 ± 0.85 ^a^
TP22	8.28 ± 1.13 ^ac^
A20	6.45 ± 0.64 ^a^
A3	6.88 ± 0.67 ^a^
T0 + 0.1 M NaCl	7.85 ± 0.85 ^a^
BacNorte + 0.1 M NaCl	11.29 ± 1.61 ^b^
J19 + 0.1 M NaCl	7.42 ± 1.80 ^a^
TP22 + 0.1 M NaCl	12.80 ± 2.91 ^b^
A20 + 0.1 M NaCl	13.98 ± 1.97 ^c^
A3 + 0.1 M NaCl	12.80 ± 1.45 ^b^

**Table 10:** Effect of the inoculation of the bacterial strain J19, TP22, A20, and A3 on the chlorophyll A content of Poncho Negro tomato plants under standard and saline conditions. Error represents the standard deviation of the five independent assays. The different letters indicate significant differences from each other (*p* < 0.05; HSD Tukey). T0 corresponds to non-inoculated tomato plants.

Treatment	[Chlorophyll A] (μg/mL)
T0	0.14 ± 0.06 ^a^
BacNorte	0.16 ± 0.14 ^a^
J19	0.16 ± 0.02 ^a^
TP22	0.24 ± 0.02 ^a^
A20	0.26 ± 0.01 ^a^
A3	0.18 ± 0.02 ^a^
T0 + 0.1 M NaCl	0.22 ± 0.04 ^ac^
BacNorte + 0.1 M NaCl	0.45 ± 0.02 ^b^
J19 + 0.1 M NaCl	0.37 ± 0.08 ^bc^
TP22 + 0.1 M NaCl	0.45 ± 0.03 ^b^
A20 + 0.1 M NaCl	0.54 ± 0.01 ^b^
A3 + 0.1 M NaCl	0.58 ± 0.09 ^b^

**Table 11:** Effect of the inoculation of the bacterial strain J19, TP22, A20, and A3 on the chlorophyll B content of Poncho Negro tomato plants under standard and saline conditions. Error represents the standard deviation of the five independent assays. The different letters indicate significant differences from each other (*p* < 0.05; HSD Tukey). T0 corresponds to non-inoculated tomato plants.

Treatment	[Chlorophyll B] (μg/mL)
T0	0.56 ± 0.10 ^a^
BacNorte	0.21 ± 0.07 ^b^
J19	0.19 ± 0.02 ^b^
TP22	0.20 ± 0.09 ^b^
A20	0.25 ± 0.03 ^b^
A3	0.10 ± 0.03 ^b^
T0 + 0.1 M NaCl	0.26 ± 0.06 ^bc^
BacNorte + 0.1 M NaCl	0.58 ± 0.11 ^a^
J19 + 0.1 M NaCl	0.41 ± 0.13 ^ac^
TP22 + 0.1 M NaCl	0.48 ± 0.18 ^ac^
A20 + 0.1 M NaCl	0.73 ± 0.13 ^a^
A3 + 0.1 M NaCl	0.46 ± 0.05 ^a^

## Data Availability

Data are contained within the article.
